# Computer-assisted analysis of functional internal rotation after reverse total shoulder arthroplasty: implications for component choice and orientation

**DOI:** 10.1186/s40634-023-00580-5

**Published:** 2023-03-14

**Authors:** Bettina Hochreiter, Michel Meisterhans, Christoph Zindel, Anna-Katharina Calek, Christian Gerber

**Affiliations:** 1 Department of Orthopedics, Balgrist University Hospital, University of Zurich, Forchstrasse 340, CH-8008 Zurich, Switzerland; 2 Balgrist Campus, Orthopaedic Research Center, Zurich, Switzerland

**Keywords:** Reverse total shoulder arthroplasty, RTSA, Internal rotation, Extension, Neck shaft angle, Baseplate, Orientation, Position

## Abstract

**Purpose:**

Functional internal rotation (IR) is a combination of extension and IR. It is clinically often limited after reverse total shoulder arthroplasty (RTSA) either due to loss of extension or IR in extension. It was the purpose of this study to determine the ideal *in-vitro* combination of glenoid and humeral components to achieve impingement-free functional IR.

**Methods:**

RTSA components were virtually implanted into a normal scapula (previously established with a statistical shape model) and into a corresponding humerus using a computer planning program (CASPA). Baseline glenoid configuration consisted of a 28 mm baseplate placed flush with the posteroinferior glenoid rim, a baseplate inclination angle of 96° (relative to the supraspinatus fossa) and a 36 mm standard glenosphere. Baseline humeral configuration consisted of a 12 mm humeral stem, a metaphysis with a neck shaft angle (NSA) of 155° (+ 6 mm medial offset), anatomic torsion of -20° and a symmetric PE inlay (36mmx0mm). Additional configurations with different humeral torsion (-20°, + 10°), NSA (135°, 145°, 155°), baseplate position, diameter, lateralization and inclination were tested. Glenohumeral extension of 5, 10, 20, and 40° was performed first, followed by IR of 20, 40, and 60° with the arm in extension of 40°—the value previously identified as necessary for satisfactory clinical functional IR. The different component combinations were taken through simulated ROM and the impingement volume (mm^3^) was recorded. Furthermore, the occurrence of impingement was read out in 5° motion increments.

**Results:**

In all cases where impingement occurred, it occurred between the PE inlay and the posterior glenoid rim. Only in 11 of 36 combinations full functional IR was possible without impingement. Anterosuperior baseplate positioning showed the highest impingement volume with every combination of NSA and torsion. A posteroinferiorly positioned 26 mm baseplate resulting in an additional 2 mm of inferior overhang as well as 6 mm baseplate lateralization offered the best impingement-free functional IR (5/6 combinations without impingement). Low impingement potential resulted from a combination of NSA 135° and + 10° torsion (4/6 combinations without impingement), followed by NSA 135° and -20° torsion (3/6 combinations without impingement) regardless of glenoid setup.

**Conclusion:**

The largest impingement-free functional IRs resulted from combining a posteroinferior baseplate position, a greater inferior glenosphere overhang, 90° of baseplate inclination angle, 6 mm glenosphere lateralization with respect to baseline setup, a lower NSA and antetorsion of the humeral component. Surgeons can employ and combine these implant configurations to achieve and improve functional IR when planning and performing RTSA.

**Level of evidence:**

Basic Science Study, Biomechanics.

## Introduction

Functional internal rotation (IR; hand on back movements) is a combined movement of extension and glenohumeral IR in extension, abduction, scapulothoracic retraction, anterior tilt and rotation [15, 30]. Maintaining or restoring functional IR with reverse total shoulder arthroplasty (RTSA) is usually not the primary treatment goal and often receives insufficient attention. A systematic review showed that in 35% of RTSA articles, IR was not reported in any form, and when it was reported, various methods were used [37]. However, functional IR accounts for approximately 10% of the Constant-Murley score (CS) and does correlate with subjective outcome [7, 16, 34]: 20% of patients have difficulty toileting after RTSA [36] and 30% are unable to wash their backs [39]. Loss of shoulder *extension* has recently been shown to be a major reason for poor functional IR after RTSA: at least 40° of extension are required for functioning of the hand behind the body. If functional IR is unsatisfactory despite 40° of extension, passive restriction of IR in full extension can be a limiting factor [17]. A possible explanation for the postoperative loss of extension and IR in extension is an impingement of the polyethylene (PE) liner on the scapular pillar [26]. Final ranges of motion (ROM) after RTSA are affected by many factors including preoperative ROM, rehabilitation, soft-tissue factors and implant position [9, 16]. Increasing impingement-free ROM improves functional outcome and reduces scapular notching [19]. Glenoid and humeral component position as well as design features significantly influence impingement-free ROM after RTSA [2, 14, 40].

So far, various biomechanical and computer modeling [10, 21–23, 25, 27, 28, 32, 33] studies have analyzed factors limiting IR, but their findings do not satisfactorily explain loss of clinical functional IR after RTSA. Most studies analyzed IR in front of the body (hand-on-abdomen movement) or in 90° of abduction [21–23], few studies analyzed extension [22, 25, 27, 40] but to date, no study has analyzed IR in ≥ 40° extension. Experimentally, a 36 mm glenosphere size with 2 mm inferior offset [25], a 135° neck-shaft angle (NSA) [22, 40] and 5–10 mm of glenoid lateralization [22, 27] seem to improve, glenoid retroversion seems to compromise extension [22].

As the surgeon has currently multiple options for implant geometry and component positioning, it was the purpose of the present study to determine the optimal combination of glenoid baseplate and humeral component position and orientation for impingement-free functional IR.

## Material and methods

### Setup and tested configurations

**Fig. 1:**
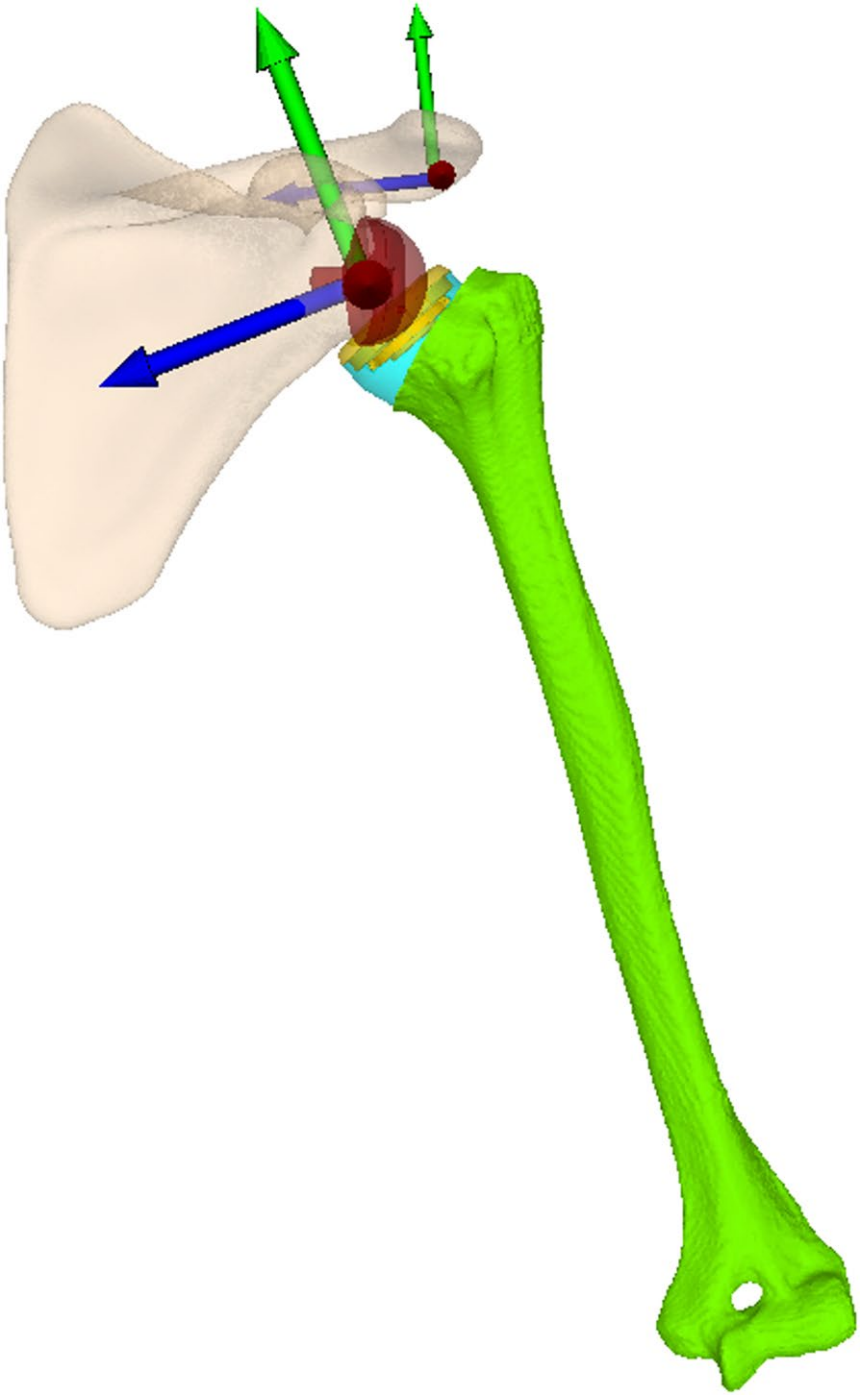
Virtual RTSA was performed on a statistical shape model of a normal scapula in a planning software (CASPA) using a 28 mm baseplate, 36 mm glenosphere, 12 mm humeral shaft and 36-0 mm polyethylene cup. For this figure a humeral cup with an NSA of 155° was chosen and the shaft was implanted in 20° of retrotorsion. The two coordinate systems of the scapula (in the back) and the humerus (in the front) according to Wu et al^41^. X-axis (mediolateral) marked in blue, y-axis in green (craniocaudal), z-axis (anteroposterior) in red. Humeral coordinate system with its center in the glenosphere in 15° glenohumeral abduction as a starting point

**Fig. 2:**
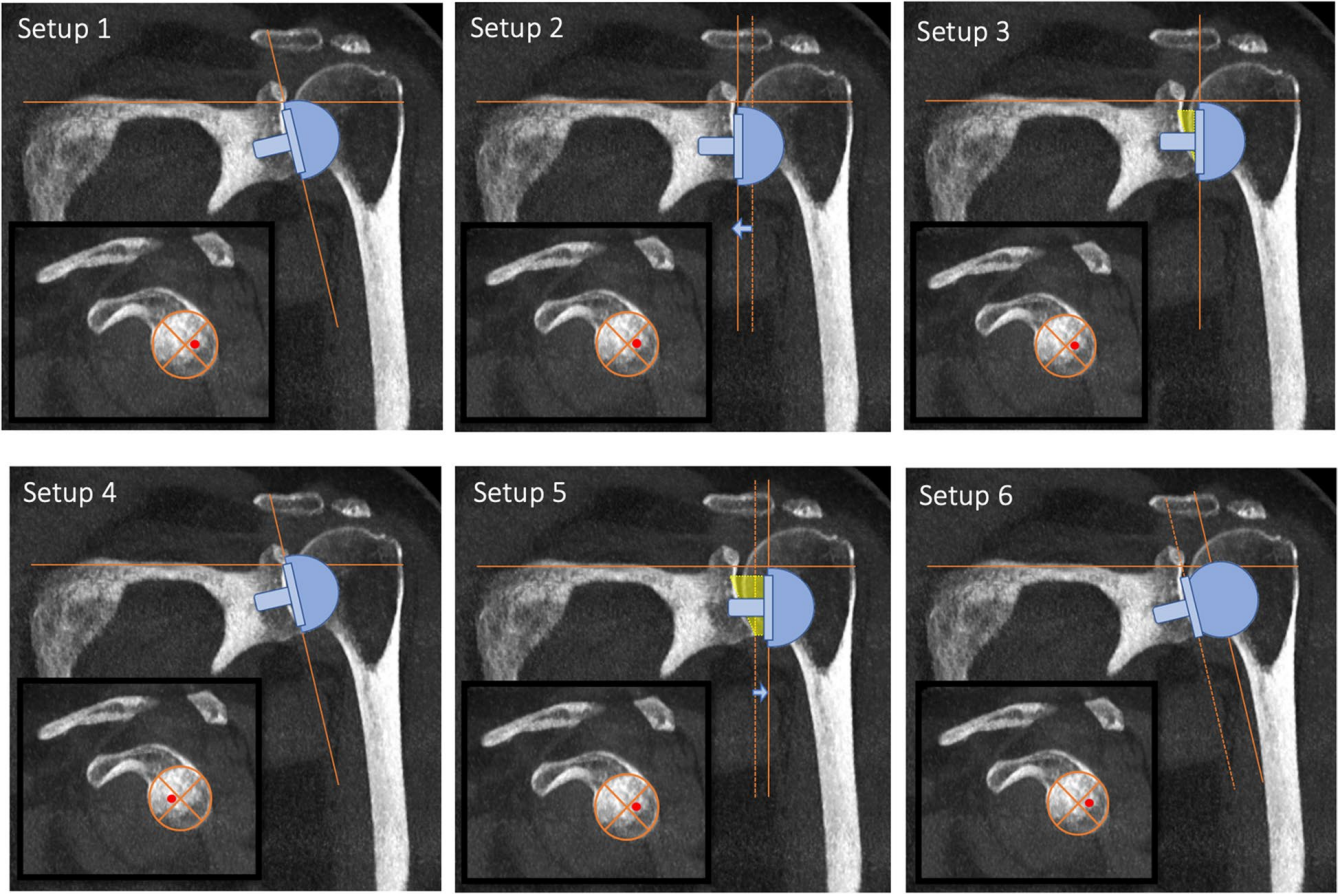
The different glenoid setups which were tested. The baseline glenoid configuration consisted of a 28 mm baseplate placed flush with the posteroinferior rim of the glenoid and a 36 mm standard glenosphere (Setup 1). Additional configurations with variable baseplate positions, lateralization and inclination were performed

**Fig. 3:**
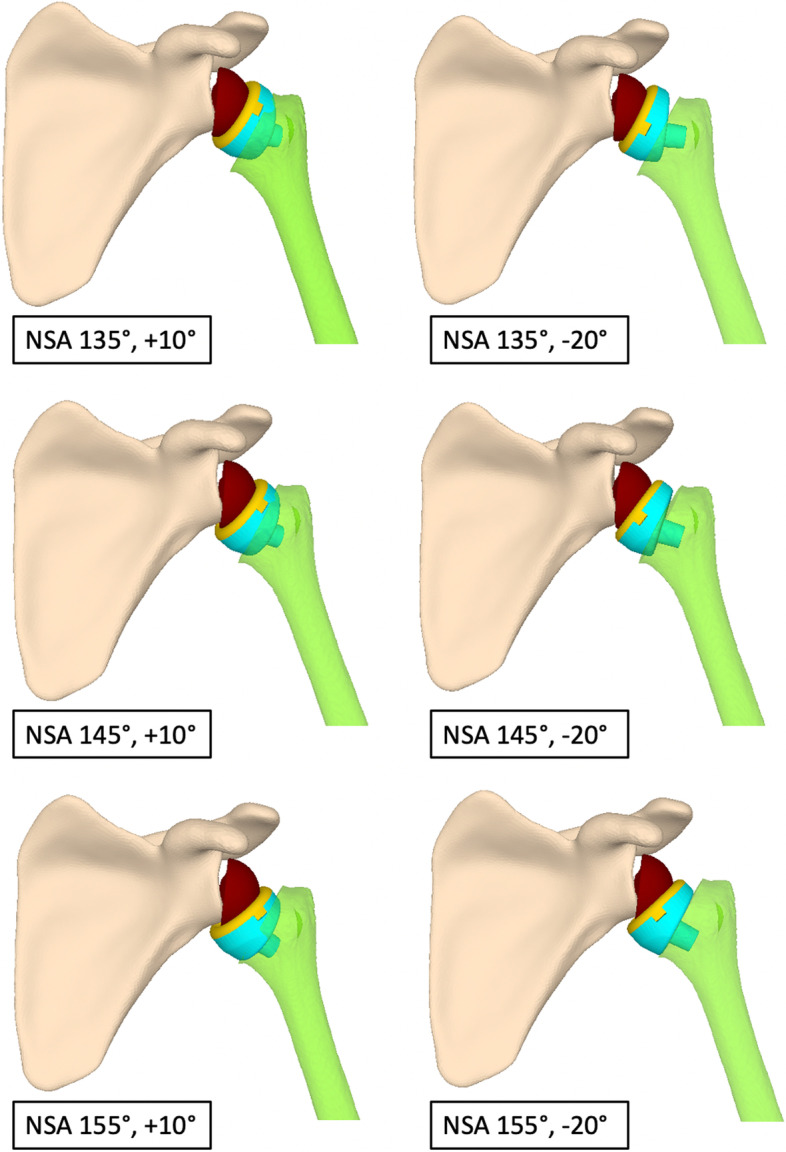
The different combinations of NSA and humeral torsion that were tested

**Fig. 4:**
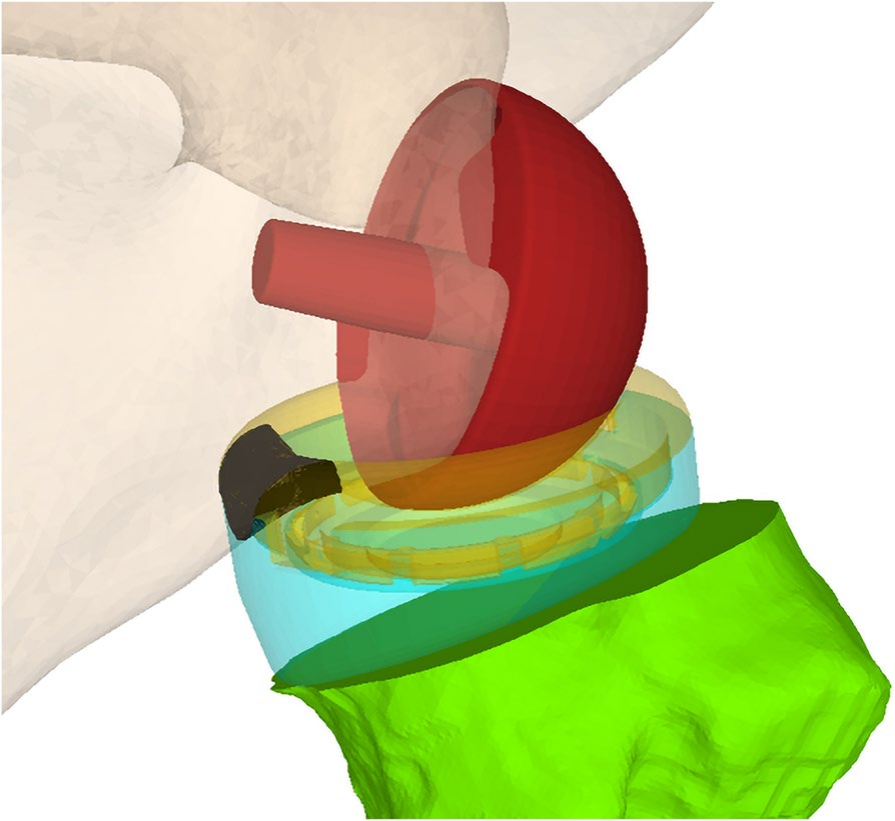
Shows the impingement between PE inlay (yellow) and posterior glenoid rim. The overlap (black) represents the impingement volume

Using the data of a previous study [1], a statistical shape model of the normal left scapula was created with computer tomography (CT) data of 40 asymptomatic shoulders. The statistical shape model was generated with a commercial software (Shapemeans, Allschwil, Switzerland) and the 3-D mean model was obtained. This mean model defined normal anatomy (-4.9° glenoid retroversion, 79.6° glenoid inclination (measured to floor of supraspinatus fossa), 31.7° Critical Shoulder Angle (CSA)). The model was then imported into the in-house developed planning software CASPA (Computer Assisted Surgery Planning Application Version 5.0) together with a 3-D left humeral model (female, 70 years) and oriented according to International Society of Biomechanics recommendations on definitions of joint coordinate systems for shoulder motions [41]. Virtual implantation of an RTSA was performed using an anatomical humeral neck cut (Fig. 1). The reference glenoid configuration consisted of a 28 mm baseplate placed flush with the posteroinferior rim of the glenoid and a 36 mm standard glenosphere (36 mm glenosphere and 15 mm peg, Trabecular Metal Reverse Shoulder System, Zimmer Biomet, Warsaw, USA). The reference humeral component configuration consisted of a 12 mm diameter uncemented humeral stem, a humeral metaphysis with an NSA of 155° (onlay design; + 6 mm medial offset), anatomic humeral retrotorsion of 20° and a symmetric PE inlay (36mmx0mm) (Anatomical Shoulder Inverse/Reverse, Zimmer Biomet, Warsaw, USA). Additional configurations with variable humeral torsion (-20° and + 10°), NSA (135°, 145°, 155°), baseplate position, glenosphere diameter, glenoid component lateralization and inclination (Figs. 2 and 3; Table 1) were performed. The baseplate inclination was calculated and reported according to Maurer [31] and modified by Boileau [3], and describes the angle between the floor of the supraspinatus fossa and the back surface of the baseplate (reverse shoulder arthroplasty angle; RSA angle). For modifications of humeral component torsion, the humerus was rotated around the coordinate system of the humeral component stem. The displacement of the baseplate was performed according to a coordinate system placed on the glenoid (Fig. 1) with the x-axis (mediolateral axis) corresponding to a 90° axis to the glenoid surface. The different NSA configurations were created by rotating the humeral cup around the x-axis of the coordinate system of the humeral cup (Fig. 4). The humerus and the scapula were oriented, so that the coordinate system of the scapula and the humerus were initially aligned to each other according to Wu et al. [41]. Glenohumeral extension corresponds to negative rotation around the x-axis (mediolateral axis), abduction corresponds to positive rotation around the z-axis (anteroposterior axis) and IR corresponds to negative rotation around the y-axis (craniocaudal axis) of the used humeral coordinate system [41]. The glenohumeral motion of an RTSA was simulated by aligning the center of the glenosphere with the center of a sphere fitted into the PE-inlay. The starting position was 15° of glenohumeral abduction [29, 30]. Glenohumeral extension of 5, 10, 20, and 40° was performed first, followed by IR of 20, 40, and 60° in the final extension position of 40°. The different glenoid and humeral configurations as well as combinations thereof were taken through simulated extension and IR and the impingement volume (mm^3^) of the scapular neck with the PE inlay was recorded with CASPA for each interposition. Furthermore, the occurrence of component impingement was read out in 5° motion increments. Impingement-free functional IR was defined as 40° extension and 60° IR.

**Table 1:** Description of the different glenoid setups that were tested

Name	Description	Transformation from Baseline Setup (No. 1)
Setup 1	posteroinferior flush baseplate on glenoid, 96° RSA angle	Baseline Setup
Setup 2	medialization and inferior tilt	3 mm medialization, 90° RSA angle (6° inferior tilt)
Setup 3	lateralisation and inferior tilt	3 mm lateralization, 90° RSA angle (6° inferior tilt) obtained with a wedge-shaped bone graft placed superiorly behind the baseplate
Setup 4	anterosuperior offset	2 mm anterosuperior, 96° RSA angle
Setup 5	BIO-RSA	6 mm lateralization, 90° RSA angle (6° inferior tilt)
Setup 6	Simulation of AltiVate (DJO Global)	6 mm lateralization, 1 mm inferoposterior (baseplate diameter 26 mm instead of 28 mm Zimmer BF), 96° RSA angle

### Statistical Analysis

Since the generated data are exact data collected by a computer system without measurement repetitions, a statistical analysis was superfluous. The data were analyzed and are reported descriptively.

## Results

### *Impingement matrix (Table **2**)*

**Table 2:** The Impingement matrix indicates the degree of extension, or combined extension/internal rotation, at which impingement occurs. The starting position is 15° abduction. From there, the humerus was extended in 5° increments to 40°. Subsequently, internal rotation was performed in this position in 5° increments. Fields with a red background indicate that impingement already occurred in the initial position of 15° abduction. Yellow fields indicate that at least a certain degree of extension was possible before impingement occurred and blue fields indicate that a certain degree of combined extension and internal rotation in extension was possible. Green fields indicate that impingement did not occur even in full internal rotation in extension

	**impingement of humerus and scapula depending on humeral component Neck-Shaft-Angle and Torsion**					
Baseplate Setup	**155° / -20°**	**155° / + 10°**	**145° / -20°**	**145° / + 10°**	**135° / -20°**	**135° / + 10°**
Setup 4	15° abduction	15° abduction	15° abduction	15° abduction	10° extension	15° abduction
Setup 2	15° abduction	15° abduction	10° extension	15° abduction	40° ext + 5° IR	25° extension
Setup 1	5° extension	15° abduction	30° extension	15° extension	40° ext + 45° IR	no impingement
Setup 3	20° extension	5° extension	40° ext + 35° IR	40° ext + 55° IR	no impingement	no impingement
Setup 5	35° extension	20° extension	40° ext + 55° IR	no impingement	no impingement	no impingement
Setup 6	40° ext + 30° IR	no impingement	no impingement	no impingement	no impingement	no impingement

In all cases where impingement occurred, it occurred between the PE inlay and the posteroinferior glenoid rim (Fig. 4). In 9 of 36 combinations, impingement already occurred in the starting position of 15° abduction (Table 2). In 10 of 36 combinations, extension between 5° and 35° was possible, but extension of 40° was not possible without impingement. In 8 of 36 combinations, full glenohumeral extension of 40° and IR between 5° and 60° was possible. Only 9 of 36 combinations allowed full IR in extension without impingement. The combination of 155° NSA and 10° antetorsion resulted in impingement in the starting position in 3 of 6 glenoid setups and allowed IR in 35° of extension in only one setup. In contrast, the combination of 135° NSA and 20° retrotorsion did not lead to impingement in the starting position in any of the cases and allowed full extension with some degree of IR in extension in 5 of 6 glenoid setups.

### Influence of glenosphere position, Neck-Shaft-Angle and humeral torsion on ROM

Glenoid Setup 6 (+ 6 mm of lateralization, smaller baseplate diameter of 26 mm, RSA angle 96°; 5/6 combinations without impingement) had the greatest effect on impingement-free functional IR, followed by a combination of NSA 135° and 10° antetorsion (4/6 combinations without impingement), followed by NSA 135° and -20° torsion (3/6 combinations without impingement) regardless of glenoid setup (Table 3). With Setup 2 and 4 impingement-free extension was not possible regardless of NSA and torsion. Setup 4 (anterosuperior baseplate offset) showed the highest impingement volume with every combination of NSA and torsion. Interestingly, comparison of Setup 1 (posteroinferior baseplate position, 96° RSA angle) versus Setup 2 (posteroinferior baseplate position, 90° RSA angle with inferior reaming) showed less impingement in 5 out of 6 combinations for Setup 1. Comparison of Setup 2 (posteroinferior baseplate position, 90° RSA angle with inferior reaming) versus Setup 3 (posteroinferior baseplate position, 90° RSA angle with superior wedge) showed significantly reduced impingement volume with all stem combinations with Setup 3 (Table 3). There was no impingement at all in 3 combinations with Setup 3 compared to one combination with Setup 2. However, Setup 5 (posteroinferior baseplate position, 90° RSA angle with lateralization – BIO-RSA) led to even less impingement volume compared to Setup 2 and 3. Comparing Setup 1 and 4 with an RSA angle of 96° and no lateralization but differences in baseplate positioning, it becomes evident that the further posteriorly and inferiorly the baseplate is positioned the less impingement volume results with extension and IR. + 10° antetorsion was more compatible with impingement-free extension/IR than -20° retrotorsion.

**Table 3:**
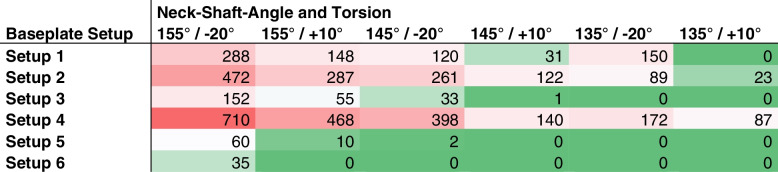
Impingement volume in the final position of 40° of extension and 60° of internal rotation

## Discussion

We used a 3-D computer model to test RTSA component combinations to simulate ROM and identify bony impingement limiting functional IR (= extension + IR). We then recombined the parameters to identify viable options to improve functional IR.

The most important result was that bony impingement in functional IR is inevitable when either the non-lateralized baseplate is not flush posteroinferiorly (= little to no glenosphere overhang) *or* a humeral stem with an NSA of 155° in 20° of retrotorsion is used. With the baseplate positioned anterosuperiorly, impingement often already occurs in the initial position of 15° abduction or only slight extension. If – for whatever reason – an NSA of 155° in 20° or retrotorsion is used, severe bony impingement can only be prevented and some functional IR can be restored with a lateralizing glenosphere and increased inferior overhang (AltiVate ® DJO Global).

Multiple, different implant geometries and implant orientation recommendations for RTSA are currently available. It appears that some are associated with less notching and at least no inferior clinical results than others. One often somewhat neglected assessment parameter is functional IR. If other clinical parameters are not adversely affected by the choice of a specific implant geometry, it would be desirable to determine, which of these are at least experimentally optimal to also restore functional IR.

The most protective parameter against impingement during functional IR is reducing the NSA from 155 to 135°; however, currently an NSA of 155° is most widely used [24]. While an impingement-free increase of ROM with decreasing NSAs has been documented, concerns regarding a potentially higher instability rate have been raised [6, 24]. A meta-analysis [11] of 2222 RTSAs compared the rate of scapular notching and instability between implants with NSAs of 155° and 135°. Scapular notching was significantly more prevalent with 155° (16.8% vs. 2.8%; *p* < 0.0001) but there was no difference in the instability rate between the two groups (2.33% for the 155° and 1.74% for the 135° group).

Looking at the humeral component a consensus regarding the torsion best suited to prevent impingement is still missing: In this study, humeral stem antetorsion was more compatible with impingement-free functional IR than retrotorsion. This is in line with previous data, however, at the expense of external rotation and overall, clinical function [18, 20]. Therefore, we do not think that increasing humeral torsion to + 10° is likely to provide increased patient benefit.

At the glenoid, a posteroinferior positioning of the baseplate is a prerequisite for impingement-free functional IR. Our data is in line with numerous other studies which have shown decreased notching and improved clinical results with distalization [32, 35] and lateralization of the glenoid [4, 8, 12]. However, lateralization alone seems insufficient to provide impingement-free ROM. Recently, Boileau et al. [4] showed that there are three risk factors significantly associated with scapular notching despite angled bony increased offset (BIO)-RTSA: (1) a low BMI, (2) an insufficient glenosphere inferior offset and (3) persistent superior inclination (RSA angle > 5°). An inferior glenosphere offset of 5 mm does obtain enough clearance below the glenosphere for the humeral cup. In our study, however, the maximum inferior glenosphere offset corresponded to 4 mm in 5 of 6 setups. Despite correct postero-inferior baseplate positioning, impingement occurred with higher NSAs. Only in setup 6, in which the inferior glenosphere offset reached 5 mm, there was no impingement despite a high NSA. Therefore, the inferior overhang must be greater than 4 mm to ensure impingement-free ROM if a 155° NSA is used. According to our data, the correction of the inferior tilt in terms of an RSA angle of 90° is only beneficial if the tilt is obtained with a superiorly placed bone graft behind the baseplate and not by inferior reaming. If a 155° humeral component is used, posteroinferior baseplate positioning without inferior reaming and an inferior glenosphere offset of > 4 mm seem to be compulsory to restore impingement free functional IR. Surgeons using a humeral component with a lower NSA have more leeway with regard to baseplate placement, but should still avoid an anterosuperior offset of the central guide wire. Only a lateralizing glenosphere (AltiVate ® DJO Global, setup 6)—even with a superior tilt of the baseplate—does not limit functional IR regardless of NSA.

Our data suggest that impingement – free functional IR does depend on choice and position of implants. At least in this experimental setup, in which only two of many implants available were tested, impingement – free passive ROM results from the following combinations:

- 145° NSA, -20° torsion, posteroinferior baseplate placement, lateralizing glenosphere with 5 mm inferior overhang (Setup 6)
- 135° NSA, -20° torsion, posteroinferior baseplate placement, lateralizing glenosphere with 5 mm inferior overhang(Setup 6)
- 135° NSA,—20° torsion, posteroinferior baseplate placement, 3 mm lateralization and 90° RSA angle obtained with a wedge-shaped bone graft placed superiorly behind the baseplate (Setup 3)
- 135° NSA, -20° torsion, posteroinferior baseplate placement and BIO-RSA (6 mm lateralization, 90° RSA angle) (Setup 5)

### Limitations

First, the study was performed on a computer model without soft tissues. Therefore, the actual clinical IR is difficult to predict with the tested implant combinations. However, passive (bony) impingement-free IR must be possible before soft tissue factors can be considered.

Secondly, the computer model is based on scapulo-humeral movements. In the simulation the scapula is fixed. In-vivo, the coupled scapular movements all contribute to IR. We therefore think that our study overestimates the necessary gleno-humeral ROM to obtain free functional IR. Nonetheless, the clinically documented limitations of ROM and the notching most frequently reported in 155° humeral components show, that scapulo-humeral impingement should be avoided and our study shows that the surgeon has the potential to do so.

A third limitation concerns the use of a SSM model of a normal scapula for the analysis. It is likely that the individual anatomy and particularly the individual length of the scapular neck has an influence on the impingement-free ROM for extension, IR and ER [38]. Furthermore, degenerative changes, especially glenoid deformity and wear, were not considered.

Fourth, we focused on extension and IR in extension because numerous other studies have already tested the influence of component positioning and choice on ROM in front and at the side of the body. However, the "ideal" component configuration for extension and IR seems to also favor ER, flexion, and abduction [4, 25, 32]. Lädermann et al. showed that with a lower NSA, increasing lateralization and, through an inferior glenosphere offset, also passive external rotation is increased.

Fifth, our data suggest that lateralizing and inferiorizing designs and positioning favor functional IR. However, it should be noted that traction on the deltoid muscle increases with lateralization and inferiorization and, accordingly, the risk of scapular spine or acromion fractures increases, as does the risk of nerve traction damage.

Sixth, the number of available implants is constantly growing and we have only tested the most common options. Further objective clinical evaluations of the implant configurations discussed in this study are needed to determine the extent to which inferiorization or lateralization also result in a clinical improvement.

## Conclusion

For the components and combinations thereof used in this experimental setup, the largest impingement-free functional IRs were seen combining a posteroinferior baseplate position, a greater inferior glenosphere overhang, 90° of baseplate inclination angle obtained with a wedge-shaped bone graft placed superiorly behind the baseplate, 6 mm glenosphere lateralization with respect to the baseline setup, lower NSA and antetorsion of the humeral component. Surgeons can employ and combine these currently available implant configurations to achieve and improve functional IR when planning and performing RTSA.

## Data Availability

The authors confirm that the data supporting the findings of this study are available within the article. Raw data are available from the corresponding author, upon reasonable request.
